# Episodic future thinking predicts differences in delay discounting: The mediating role of hippocampal structure

**DOI:** 10.3389/fpsyg.2022.992245

**Published:** 2022-10-12

**Authors:** Yiqun Guo, Huimin Wu, Zhangyong Li, Le Zhao, Tingyong Feng

**Affiliations:** ^1^School of Innovation and Entrepreneurship Education, Chongqing University of Posts and Telecommunications, Chongqing, China; ^2^School of Bioinformatics, Chongqing University of Posts and Telecommunications, Chongqing, China; ^3^Key Laboratory of Cognition and Personality, Ministry of Education, Chongqing, China; ^4^Faculty of Psychology, Southwest University, Chongqing, China; ^5^Faculty of Psychology, Beijing Normal University, Zhuhai, China

**Keywords:** episodic future thinking, delay discounting, VBM, hippocampus, mediation

## Abstract

A growing body of evidence suggests that engagement in episodic future thinking (EFT) could reduce delay discounting rates. However, little is known about whether individual differences in the ability of EFT are associated with differences in delay discounting in young adults. In the present study, this association was tested in healthy college students (*n* = 106, 19.98 ± 1.56 years), and the neural basis underlying this association was also examined using voxel-based morphometry (VBM) method. Behavioral analysis indicated that individual differences in EFT ability can significantly negatively predict discounting rates. VBM analysis first revealed that the EFT score positively correlated with gray matter volume (GMV) of a cluster in hippocampus, while negatively correlated with GMV of a cluster in rostral anterior cingulate cortex. We also found the GMV of a cluster in the mPFC was positively correlated with delay discounting. ROI analysis further revealed that individual differences in delay discounting could be reliably predicted by the GMV in the hippocampus and mPFC. The final mediation analysis showed that the GMV of the hippocampus plays a significant mediating role in the association between EFT and delay discounting, and the indirect effect of the hippocampal GMV accounts for 33.2% of the total effect. Our results suggest that individuals’ EFT ability may be an important determinant of differences in delay discounting, and highlight the hippocampal structure as a neural biomarker for explaining the association between EFT ability and delay discounting.

## Introduction

Although humans have the ability to anticipate the future consequences (Suddendorf and Busby, 2005), we often devalue future rewards when making intertemporal choices, a phenomenon known as delay discounting (Frederick et al., 2002). Discounting rates are fairly stable across time with an individual (Kirby, 2009; Odum, 2011), but vary widely cross individuals (Frederick et al., 2002). A large body of evidence has suggested that delay discounting can act as a behavioral marker of impulsivity and steeper discounting rates are associated with a number of maladaptive behaviors such as, substance abuse, pathological gambling, obesity (Bickel and Marsch, 2001; Dixon et al., 2003; Amlung et al., 2016). Episodic future thinking (EFT) refers to mental time travel forward in time to pre-experience a future event, which might be a human-specific ability that allows the construction of episodic details for future events and integration of those details with higher-order autobiographical knowledge (Atance and O’Neill, 2001). It has proven to be a promising approach reducing the bias towards immediate gratification or delay discounting (Peters and Büchel, 2010; Daniel et al., 2013a,b; Stein et al., 2016; Zhang et al., 2018; O'Donnell et al., 2019). However, there are still some questions remain unclear regarding whether individual differences in the ability of EFT are associated with delay discounting in young adults.

Episodic future thinking (EFT) is believed to reduce delay discounting by activating the mental representation of the delayed rewards and increasing the saliency and vividness, so that the future rewards are more easily accessible (Peters and Büchel, 2010; Benoit et al., 2011). In general, researchers used modified intertemporal choice tasks in which personally relevant future event cues or control conditions (i.e., no manipulation, non-episodic or episodic recent thinking) were displayed along with the reward options to investigate the effect of EFT on delay discounting (Lin and Epstein, 2014; O'Donnell et al., 2019). For example, Peters and Büchel (2010) found a significant reduction of discounting rates for the delayed reward when participants were requested to image a future event, and the extent of this reduction was associated with the vividness of the imagined event. This EFT effect has subsequently been replicated in some impulsive individuals, such as smokers intending to quit or reduce smoking (Chiou and Wu, 2017), obese children and women (Daniel et al., 2013a, 2015), people with alcohol dependence (Snider et al., 2016), and patients with hippocampal amnesia (Kwan et al., 2015).

Functional magnetic resonance imaging (fMRI) studies have revealed that the neural mechanisms of the EFT are mainly located in the in the medial temporal lobe (MTL) including the hippocampus and parahippocampal cortex, the medial prefrontal cortex (mPFC), posterior cingulate cortex, and lateral temporal and parietal regions that largely corresponds to the default mode network (Benoit and Schacter, 2015; Raichle, 2015; Stawarczyk and D'Argembeau, 2015; Schacter et al., 2017). Researchers used different paradigms and jointly found that the hippocampus was responsive to the construction of details and vividness of imagined future events (van Mulukom et al., 2013; Szpunar et al., 2014; Madore et al., 2016). A recent study conducted voxel-based morphometry (VBM) to examine the neural basis of the EFT, and results showed that the detail and vividness of future imagery was significantly correlated with regional gray matter volume (GMV) of the hippocampus and putamen (Yang et al., 2020). These findings suggest a link between hippocampus and the EFT ability. Furthermore, the MTL, especially the hippocampus, is also an important region that contributes to delay discounting (Peters and Büchel, 2010, 2011). Thus, the MTL may be a neural basis for the association between EFT and delay discounting.

Several studies have investigated the association between EFT ability and delay discounting, but the finding is mixed. Two previous studies found that individual differences in EFT are associated with differences in discounting in adolescents (Bromberg et al., 2015; McCue et al., 2019). Burns et al. (2021) found a weak correlation between EFT ability and performance on delay discounting in children aged 7–11 years, but the correlation did not survive after controlling for age and IQ. These inconsistent findings may be because the EFT ability itself continues to develop from childhood to adolescence, and children require more effort to engage in EFT than adolescents. Therefore, the present study used a sample of young adults to further test whether there was a significant association between EFT ability and delay discounting.

Neuroanatomical structure can reflect individual differences in various cognitive processes (Gaser and Schlaug, 2003; Gilaie-Dotan et al., 2011; Song et al., 2011; Kong et al., 2015), also including delay discounting (Guo et al., 2017; Owens et al., 2017; Lempert et al., 2020). Delay discounting has been found to be associated with structural features of brain regions involved in EFT such as the MTL and middle temporal gyrus (Owens et al., 2017; Lempert et al., 2020; Garzón et al., 2022). Here, we utilized the VBM method to uncover the prediction of the EFT on delay discounting. We first adapted a widely-used EFT paradigm to assess individual differences in the ability of EFT which refers to the perceived vividness and other phenomenological qualities of imagined events and examine its association with delay discounting (D’Argembeau and Van der Linden, 2012; Yang et al., 2020). We expected that individuals with a high level of EFT ability would prefer delayed rewards in intertemporal choice task, and this preference may be associated with brain structure of the EFT, especially the MTL.

## Materials and methods

### Participants

The sample consisted of 118 healthy participants from Southwest University (China) who volunteered to participate in this study. 12 participants were excluded for further analysis because of either missing data (six participants with missing EFT or fluid intelligence data) or incomplete MRI scanning (6 participants), leaving 106 participants (78 females, age, 19.98 ± 1.56 years). Prior to the study onset, all participants signed an informed consent document, and none reported a history of neurological or psychiatric disorder. The experimental protocol was approved by the Ethics Committee of the Southwest University.

### Experimental procedure

Before the MRI scanning, all participants were required to complete the behavioral measures used to characterize individual EFT ability and delay discounting. In order to avoid a possible carry over effect from EFT task to the delay discounting task, we asked participants to first complete the delay discounting task and then the EFT task.

### Assessment of fluid intelligence

To control for the possible effect of fluid intelligence on the relation between EFT and delay discounting (Shamosh and Gray, 2008; Nusbaum and Silvia, 2011; Burns et al., 2021; Frith et al., 2021), we assessed the participants’ fluid intelligence using the Raven’s Standard Progressive Matrices for adults (Raven and Court, 1998), which is a global nonverbal measure of abstract reasoning and has been proven to have good reliability and validity in Chinese sample (Zhang, 1989). It consists of 60 items, in which participants were shown a matrix of pictures with missing parts and asked to select one of several possible answers that matches the visual features of the pictures. The number of correctly answered items was used to measure participants’ fluid intelligence.

### Delay discounting

**Figure 1:**
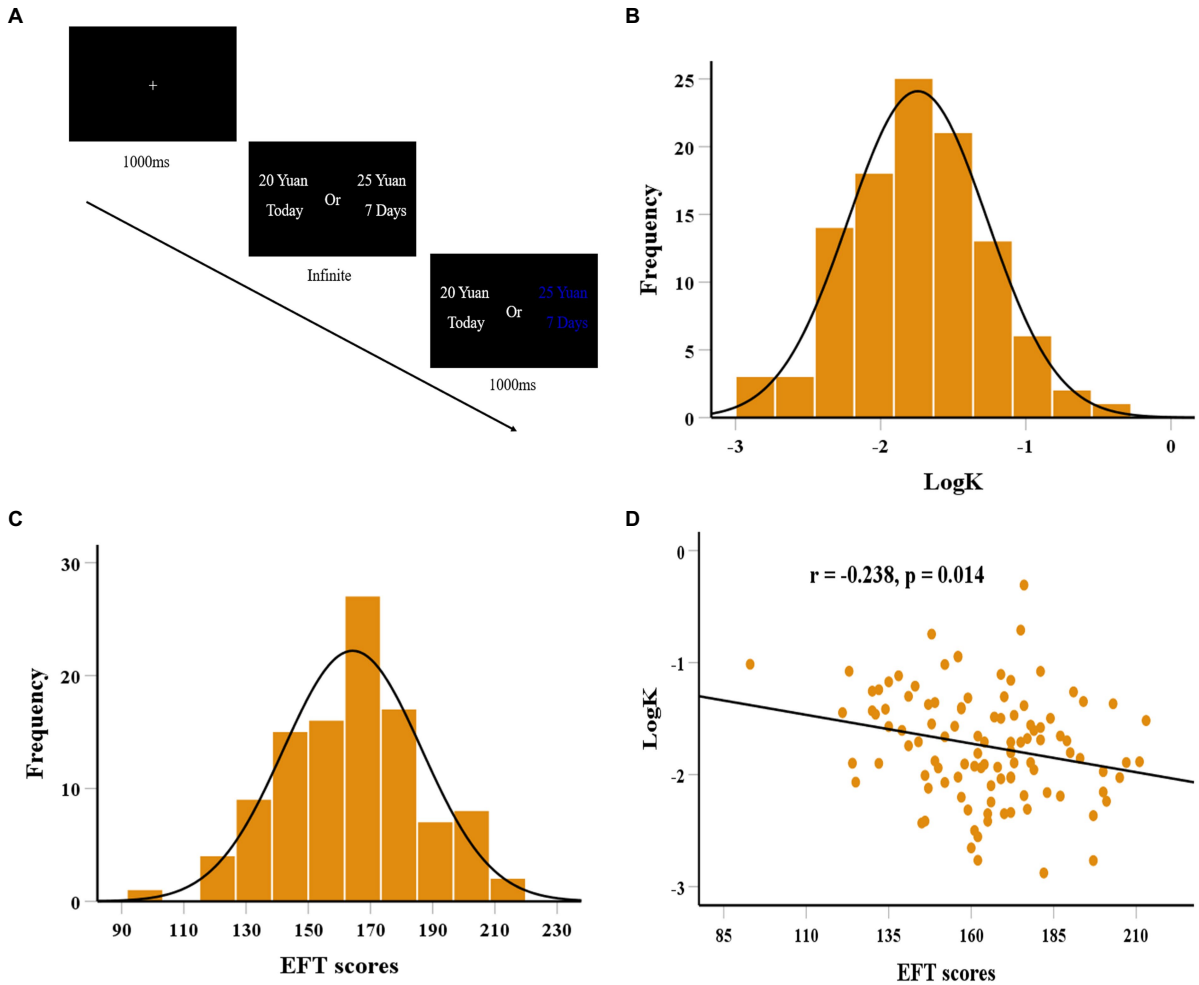
The flow chart for the delay discounting task **(A)**. The distributions of logK **(B)** and EFT scores **(C)**. Scatter plot shows the correlation between episodic future thinking (EFT) ability and logK **(D)**.

We administered a monetary delay discounting task in which participants were required to complete a total of 70 choices between an immediate reward and a delayed reward (see Figure 1A). In accordance with previous studies (Reynolds and Schiffbauer, 2004; Yi et al., 2017; DeHart et al., 2020), the delayed reward was always CNY100 presented at one of five delays (7, 15, 30, 60, 120 days). Delay periods were presented in blocks of 14 trials per delay. The immediate rewards varied from CNY 3 to CNY 99 for the first 10 of 14 trials in each delay block. Next, in order to accurately estimate the indifference point, for 4 of the 14 trials, the reward size of the immediate reward was adjusted and around at the indifference point based on the subject’s decisions in the previous trials (van den Bos et al., 2014). At the end of the experiment, participants randomly selected one trial to be paid as the reward for their remuneration.

For each individual, logistic functions were firstly used to fit the participant’s choices, and quantified an indifference point (a point of subjective equality for immediate reward and delay reward, *p* = 0.5) at each delay (Wileyto et al., 2004; Guo et al., 2017). Then, a hyperbolic function of the form SV = 1/(1 + *k*D) (SV, subjective value; D, delay in days; *k*, discount rate) has been utilized to fit one’s choice to obtain discount parameters (*k*) using maximum likelihood estimation (Peters and Büchel, 2010; van den Bos et al., 2014). Finally, we log-transformed the discount rate (logk) given that the *k* estimates were not normally distributed. Higher logk values represent steeper discounting rates.

### Episodic future thinking measures

We used a questionnaire adapted from a paradigm developed by D’Argembeau and Van der Linden (2012) to assess individual differences in the ability of EFT (Yang et al., 2020). Participants were instructed to imagine a series of specific future events that occur at least 1 year later based on cue words. To make future events easy to imagine, all cue words were drawn from familiar people or daily activities (e.g., family, study, friend, party, and trip) and the order of the cues was counterbalanced across participants (D’Argembeau and Van der Linden, 2012; Rebetez et al., 2016; Yang et al., 2020). Participants were asked to imagine a single event and keep the imagination in mind for more than 1 min, ensuring that each participant fully imagined future events. After each imagination, participants needed to complete a 7-point Likert-type questionnaire to assess the characteristics of their future thoughts (D’Argembeau and Van der Linden, 2012). The first five items evaluated the details and vividness about future imagination overall including vividness, amount of visual and other sensory details, clarity of location, persons, and objects, and the other two items evaluated the subjective consciousness (mentally traveling to the time when the event would happen) and the feeling of pre-experiencing the event. We calculated the summed scores for all the events as the ability of EFT, with higher scores on this measure indicate greater EFT ability. This questionnaire has been used to investigate the neural correlates of EFT in previous study, and has proven to be a good way to assess the individual differences in EFT (Yang et al., 2020). The Cronbach alpha coefficient for this measure of EFT ability was 0.91 in the present sample.

### Magnetic resonance imaging structural acquisition

High-resolution anatomical images were acquired with a Siemens 3T scanner (Siemens Magnetom Trio TIM, Erlangen, Germany). Participants were required to have a rest and keep their heads still during scanning. T1-weighted structural images (1 × 1 × 1.33 mm^3^) were acquired with an MPRAGE pulse sequence (128 slices; TR = 2,530 ms; TE = 3.39 ms; flip angle = 7°; 256 × 256 matrix).

### Voxel-based morphometry analysis

We conducted VBM analysis to quantify the GMV of regions using SPM12 (Wellcome Trust Centre for Neuroimaging)^1^ and Computational Anatomy Toolbox (CAT12).^2^ Each structural image was checked for artifacts and gross anatomical abnormalities and manually reoriented using the “Display” option so that the coordinate of the anterior commissure matched the origin, and the orientation approximated MNI space. Next, structural MR images were classified into gray matter, white matter (WM) and cerebrospinal fluid (CSF) using CAT12 segmentation tool. Segmented GM maps were warped to the standard Diffeomorphic Anatomical Registration Through Exponentiated Lie Algebra (DARTEL) template and normalized to the Montreal Neurological Institute (MNI) space. Normalized GM maps were then modulated to obtain the volume of GM tissue corrected for individual brain sizes, and smoothed with an 8 mm Gaussian FWHM. The homogeneity check showed no outliers.

In group analysis, we performed two multiple regression models to identify regional GMV that correlated with the ability of EFT or delay discounting. The covariate of interest included in the model was the EFT scores or logk, while age, gender, fluid intelligence, and the total GMV of the participants were also included as covariates. An absolute threshold for masking of 0.2 was used to exclude voxels outside of the brain. T contrasts were applied to detect voxels with significant correlation to the ability of EFT or delay discounting. Statistical parametric maps were subsequently corrected for multiple comparisons using Gaussian random field theory with a voxel level of *p* < 0.001 and a cluster level of *p* < 0.05.

### Cross-validation analysis

To identify the robustness of the relations among EFT, delay discounting, and regional GMV, we adopted a fourfold balanced cross-validation approach combined with linear regression (Cohen, 2010; Qin et al., 2014). First, a dependent variable and an independent variable were input into the linear regression algorithm. The *r*_(predicted, observed)_ that represented the prediction of the independent variable on the dependent variable was estimated using a balanced four-fold cross-validation procedure. First, the data were divided into four-fold so that the distribution of these variables was balanced in each fold. Second, we built a linear regression model using the three folds, leaving out the fourth fold, to predict the data in the left-one fold (i.e., predicted values). This procedure was repeated four times to obtain a final *r*_(predicted, observed)_, which represented the association between the observed data and the data predicted by the regression model. Here, nonparametric testing method was used to assess the statistical significance of the regression models. The empirical null distribution of *r*_(predicted, observed)_ was estimated by generating 1,000 surrogate datasets based on the null hypothesis that there was no association between delay discounting and EFT scores. Each surrogate dataset Di was generated by reassembling the labels on the observed data points with a size equal to the observed dataset. Then, using the observed labels of Di and prediction labels, the *r*_(predicted, observed)_ *i* was calculated using the four-fold-balanced cross-validation procedure described above. The statistical significance (value of *p*) of the model was determined by counting the number of *r*_(predicted, observed)_ *i* greater than *r*_(predicted, observed)_ and then dividing that count by the number of Di data sets (i.e., 1,000).

## Results

### Behavioral results

We first examined the distribution of behavioral data indicating the normality of logk (Kolmogorov–Smirnov *z* = 0.054, *df* = 106, *p* = 0.200; see Figure 1B) and EFT (Kolmogorov–Smirnov *z* = 0.044, *df* = 106, *p* = 0.200; see Figure 1C). No gender differences were found for EFT (*t* = 1.032, df = 104, *p* = 0.304) and logk (*t* = 0.908, *df* = 104, *p* = 0.366). There were also no significant correlations between EFT and age (*r* = 0.053, *df* = 104, *p* = 0.587), and between logk and age (*r* = 0.044, *df* = 104, *p* = 0.654). Of note, EFT was negatively correlated with logk (*r* = −0.238, *df* = 104, *p* = 0.014; see Figure 1D). regression analysis further found that individual differences in EFT could significantly predict logk after controlling for age, gender, and fluid intelligence (*b* = −0.257, *t* = 2.679, *df* = 101, *p* = 0.009). To examine the robustness of the relation between EFT ability and delay discounting, a balanced fourfold cross-validation approach combined with linear regression was conducted. We found that individual differences in delay discounting were reliably predicted by the standardized residuals for the EFT scores after regressing out age, gender, and fluid intelligence (*r*_(predicted, observed)_ = 0.227, *p* = 0.010), suggesting the robust association of EFT ability with delay discounting.

### Results of VBM analysis

**Figure 2:**
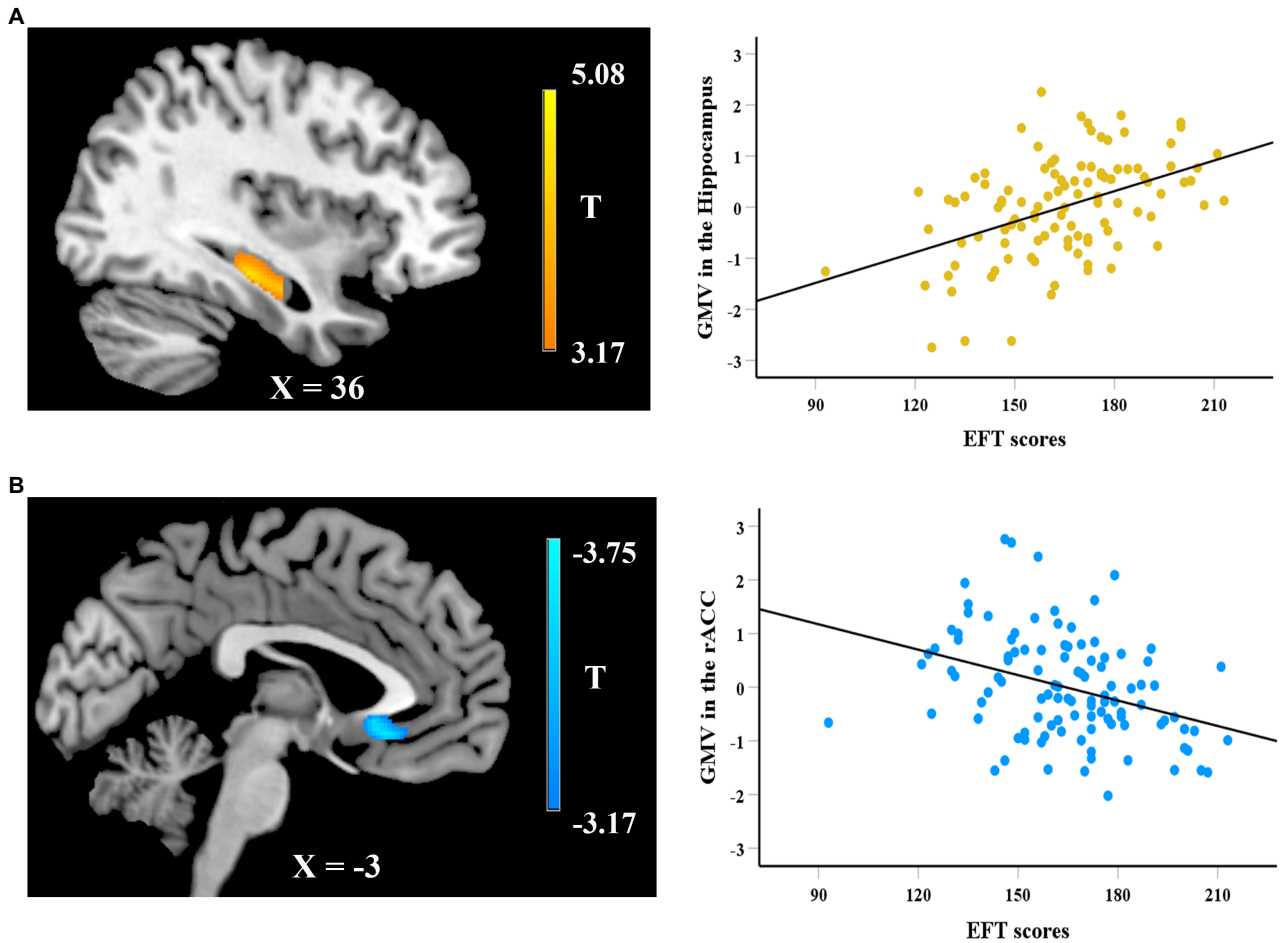
Whole-brain regression analysis showed that the gray matter volume (GMV) of a cluster in hippocampus was positively correlated with EFT scores **(A)**, while a cluster in rACC was negatively correlated with EFT scores **(B)**. Scatter plots on the right side are presented for visualization purposes only and not to be used for statistical inference.

**Figure 3:**
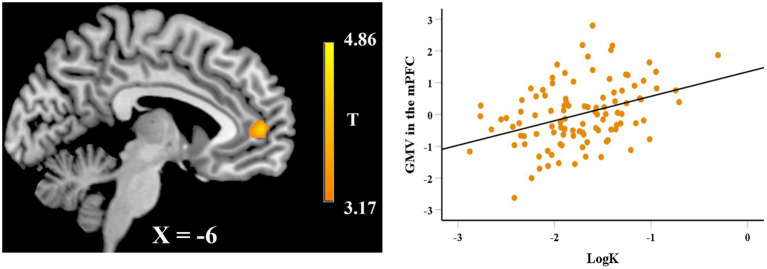
Whole-brain regression analysis showed that delay discounting (logk) was significantly correlated with regional GMV in the mPFC. Scatter plot on the right side is presented for visualization purposes only and not to be used for statistical inference.

In the VBM analysis, we first explored the neuroanatomical correlates of EFT. After adjusting for gender, age, fluid intelligence, and total GMV, whole-brain regression analysis showed that the GMV of a cluster in hippocampus (peak MNI coordinate: 37.5, − 24, − 15; 183 voxels) was positively correlated with the EFT scores (see Figure 2A), while the GMV of a cluster in rostral anterior cingulate cortex (rACC, peak MNI coordinate: 0, 25.5, −9; 356 voxels) was negatively correlated with EFT scores (see Figure 2B). Whole-brain regression analysis also found that the GMV of a cluster in mPFC (peak MNI coordinate: −10.5, 52.5, 6; 310 voxels) was positively correlated with logk (see Figure 3).

**Figure 4:**
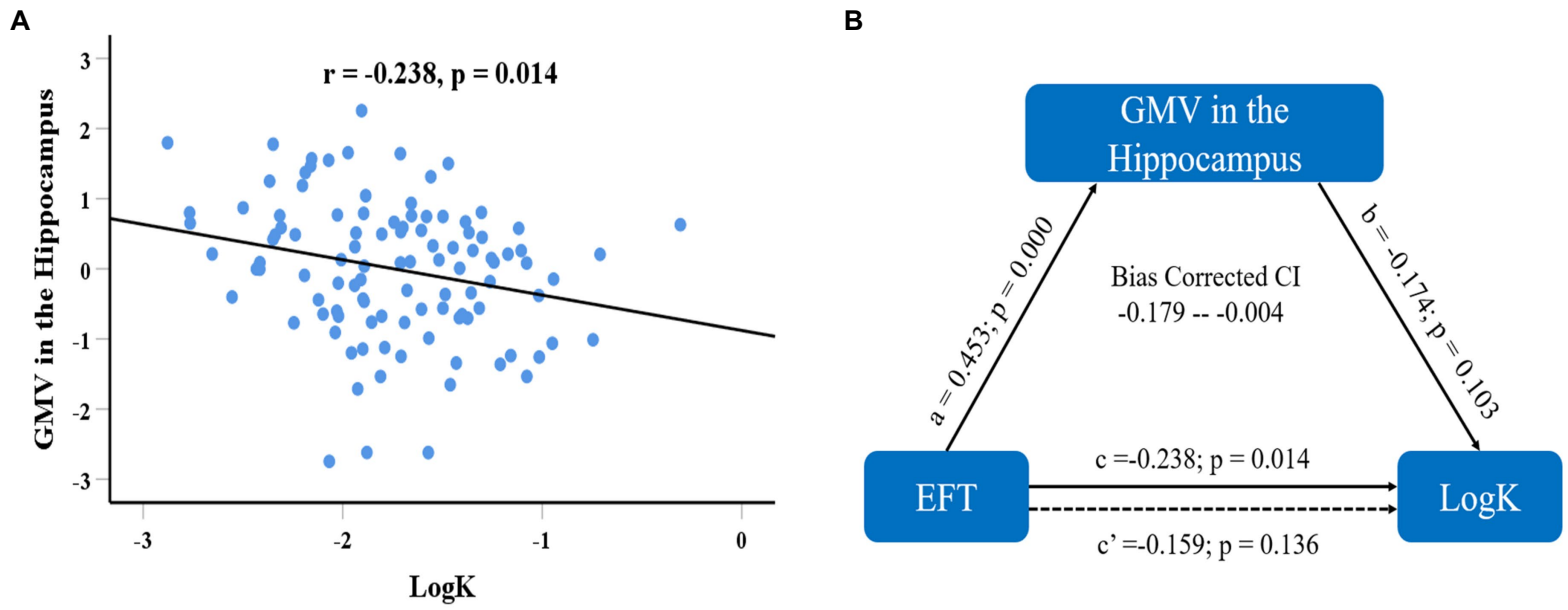
Scatter plot shows the correlations of delay discounting with regional GMV in the hippocampus **(A)** after adjusting age, sex, fluid intelligence, and total GMV. Mediation analysis showed that GMV of the hippocampus mediated the effect of EFT ability on delay discounting **(B)**, and the indirect effect accounts for 33.2% of the total effect [(*c*–*c*′)/*c*].

We first defined the brain regions in which GMV was correlated with EFT as regions-of-interest (ROIs) and calculated the mean value of GMV within each ROI. ROI analyses first examined whether the brain structures correlated with EFT were associated with delay discounting. After regressing out age, gender, fluid intelligence, and total GMV, standardized residuals for the GMV in each ROI were computed and used in the subsequent analyses. Correlation analyses showed that the GMV in the hippocampus that was associated with EFT was negatively correlated with logk (*r* = −0.246, *df* = 104, *p* = 0.011; see Figure 4A), while the correlation between the regional GMV in the rACC and logk was not significance (*r* = 0.175, *df* = 104, *p* = 0.072). Prediction analyses using balanced four-fold cross-validation approach were performed to examine the robustness of the association between regional GMV and delay discounting, and it was found that individual differences in delay discounting could be reliably predicted by regional GMV in hippocampus (*r*_(predicted, observed)_ = 0.235, *p* = 0.005) but not by regional GMV in rACC (*r*_(predicted, observed)_ = 0.175, *p* = 0.130). Furthermore, we also defined the mPFC that correlated with delay discounting as the ROI and extracted its mean value of GMV. However, the standardized residuals for the GMV in the mPFC after regressing out age, gender, fluid intelligence, and total GMV were not significantly correlated with EFT scores (*r* = −0.024, *df* = 104, *p* = 0.805).

We finally built a regression model to determine which regions contributed uniquely to delay discounting, in which the GMV in the mPFC, hippocampus, and rACC was entered simultaneously. Regression analysis found that the regional GMV in the mPFC and hippocampus could significantly predict delay discounting after controlling for age, gender, fluid intelligence, and total GMV (mPFC: *b* = 0.495, *df* = 98, *t* = 4.126, *p* < 0.000; hippocampus: *b* = −0.337, *t* = 2.879, *df* = 98, *p* = 0.005). These results showed that the GMV in the hippocampus was significantly associated with both EFT and delay discounting.

### Results of mediation analysis

To further characterize the association between the GMV in the hippocampus, EFT, and delay discounting, we conducted mediation analysis using the Hayes’s (2017) PROCESS macro to examine whether regional GMV in the hippocampus plausibly contributed to the association between EFT and delay discounting. The mediation model was estimated to derive the total, direct, and indirect effects of EFT ability and DD. A bootstrapping procedure with 5,000 bootstrap samples was used to estimate the indirect effect of the mediation model. The 95% confidence interval (CI) for the product of indirect path that does not include zero provides evidence of a significantly indirect effect (Hayes, 2009). The mediation analysis demonstrated a significantly indirect effect of EFT on delay discounting through regional GMV in the hippocampus (indirect effect = 0.079, 95% CI = −0.179 – −0.004; see Figure 4B). The indirect effect accounts for 33.2% of the total effect of EFT ability on delay discounting, which results in an insignificant direct effect (C′ = 0.159, *p* = 0.136). In summary, regional GMV in the hippocampus plays a mediating role in the association between EFT and delay discounting.

## Discussion

In the present study, we investigated the association between EFT and delay discounting from an individual differences’ perspective. Behavioral analysis indicated that individual differences in EFT could significantly negatively predict differences in delay discounting. It seems that people with a high level of EFT ability show less impulsivity in delay discounting. VBM analysis first revealed that regional GMV in the hippocampus was positively correlated with EFT scores, while regional GMV in the rACC was negatively correlated with EFT scores. We also found a significant correlation between regional GMV in the mPFC and discounting rates. ROI analysis further revealed that individual differences in delay discounting could be reliably predicted by regional GMV in hippocampus after adjusting participants’ age, gender, and total GMV. The final mediation analysis showed that regional GMV in hippocampus mediated the association between EFT and delay discounting, which suggests that the hippocampus might be the neural basis underlying this association.

Our results indicate that individual differences in EFT ability contribute to differences in delay discounting in young adults. Previous studies have shown that engaging in imagery of personal future events (EFT cues) during decision making can attenuate discounting rates in adults (Peters and Büchel, 2010; Benoit et al., 2011; Daniel et al., 2013a; Bromberg et al., 2017). In majority of these studies, personal future events and the delayed rewards offered were always time-locked, which provide evidence to support short-lived “state” changes in discounting behavior. However, the role of EFT cues in reducing discounting rates suggests that “trait” differences in EFT may be an important contributor in delay discounting. Bromberg et al. (2015) found a negative relation between the amount of episodic details produced in response to future thinking and delay discounting in adolescents after controlling for variables some variables such as age, IQ, episodic memory, and impulsivity. McCue et al. (2019) replicated Bromberg et al., (2015) finding in a larger sample of adolescents in older adults. In addition, Lempert et al. (2020) found a significant association between episodic memory and delay discounting. It is possible that a vivid imagination of the future depends on the ability to retrieve detailed episodic memories (Boyer, 2008; Schacter et al., 2012). Consistent with the above findings, the present study provides behavioral and neuroimaging evidence showing that EFT ability contributes to individual differences in delay discounting in adults.

Neuroimaging results revealed that regional GMV of clusters in the hippocampus and rACC significantly correlated with EFT scores, whereas regional GMV of a cluster in the mPFC positively correlated with delay discounting. The reason for the difference findings between EFT and delay discounting related regional GMV in the whole-brain analyses may be that the imagery or prospection is only one of subprocesses underlying delay discounting that contribute to differences in choices. fMRI studies have also shown that delay discounting involves the processes of reward valuation and cognitive control (for review see: Peters and Büchel, 2011). Furthermore, our ROI analyses found that individual differences in delay discounting could be reliably predicted by regional GMV in hippocampus that correlated with EFT, which provided evidence to support that episodic imagery is an important process of delay discounting.

A growing number of findings support the critical role of the hippocampus in episodic memory and episodic future thinking (for reviews see: Schacter et al., 2012, 2017). Benoit and Schacter (2015) conducted a meta-analysis of fMRI studies that examined the spatial overlap between episodic memory and episodic simulation, and identified a core network that are jointly recruited during episodic retrieval and simulation, including the hippocampus, lateral temporal and parietal cortex. Szpunar et al. (2014) used the fMRI repetition suppression paradigm to examine the contribution of the specific regions and identified distinct regions contributing to component features of event. Their results also suggested that the hippocampus was associated with event novelty, which was further confirmed in another independent fMRI study of repeated event simulations (van Mulukom et al., 2013). In their study, Madore et al. (2016) encouraged participants to recall the details of past experience during the construction of imagined future events in an episodic specificity induction procedure, and found that induction-related differences in detail of imagined events were significantly related to induction-related differences in neural activity of left anterior hippocampus and inferior parietal lobule. A VBM study further revealed the association between detail and vividness of EFT and regional GMV in the hippocampus (Yang et al., 2020). Consistent with these findings, our results highlight the association between hippocampal structure and episodic simulation of future events.

Moreover, the rACC and adjacent vmPFC have been shown to play an important role in the positive simulation of future events (Sharot et al., 2007; Blair et al., 2013; Murphy et al., 2017). Sharot et al. (2007) revealed increased activity in rACC when participants were required to imagine positive future event compared with negative future events. Murphy et al. (2017) further found that positive imagery training increased rACC activity, and rACC activity was positively correlated with the pleasantness ratings of images formed in older healthy adults. The association between EFT and regional GMV in rACC might reflect the participants’ positive bias in their expectation of future events. However, the GMV in the rACC cannot predict the individual differences in delay discounting. Although previous studies showed that engagement in positive episodic future thinking usually evokes positive prospective emotion that attenuated delay discounting rates (Liu et al., 2012; Zhang et al., 2018), this effect is typically observed when future event cues and intertemporal choices are presented simultaneously. In the present study, we asked participants to first complete the delay discounting task and then the EFT task, which avoided a possible carry over effect from positive future thinking to the delay discounting.

Our whole-brain regression analysis also found that the GMV in the mPFC was positively correlated with delay discounting, which is consistent with some previous studies (Cho et al., 2013; Wang et al., 2016; Guo et al., 2017). fMRI studies have shown that the mPFC is a core brain region of the valuation network that scales with subjective value of delayed rewards (Kable and Glimcher, 2007, 2010). Some MRI studies also found that the GMV in the mPFC was positively associated with delay discounting. One lesion study on humans that observed increases in discounting rates involved damage of the mPFC (Sellitto et al., 2010). The above studies suggest that the mPFC may be a core region that reflect individual differences in delay discounting.

Importantly, our mediating analysis showed that regional GMV in the hippocampus mediated the association between EFT ability and delay discounting. Converging evidence from our and previous studies suggests that the EFT may be an important psychological resource for fostering an individual’s future-orientation decision making (Bromberg et al., 2015; McCue et al., 2019). Furthermore, neuroimaging studies suggest that the hippocampus plays critical role in simulation of future events (for reviews see: Schacter et al., 2012, 2017), and is also an important region that contributes to delay discounting (Peters and Büchel, 2010, 2011). Previous studies have found that rodents with lesions in the hippocampus showed increased delay discounting (Cheung and Cardinal, 2005; Mariano et al., 2009), and hippocampal damage in humans can also lead to impairments in decision making (Gupta et al., 2009). However, case studies found that patients with episodic amnesia whose medial temporal lobe (includes hippocampus and parahippocampal cortex) is damaged showed normal discounting rates (Kwan et al., 2012; De Luca et al., 2018), which may be due to the small samples or the extent of damage within the medial temporal lobe. Craver et al. (2014) found that some patients with episodic amnesia seemed able to think of the future and to consider the distant outcomes of his behaviors. Clinical studies have found that patients with hippocampus atrophy due to Alzheimer’ disease had a bias toward immediate rewards and demonstrated a steeper discounting than healthy controls (Thoma et al., 2017; El Haj et al., 2020), while hippocampal neurogenesis in rats could contribute to a great preference for future rewards (Seib et al., 2021). A VBM study also found that hippocampal white matter volume correlated with delay discounting (Yu, 2012). Moreover, the hippocampus may involve in cognitive control and adaptive behavior *via* its connection with prefrontal cortex (Jiang et al., 2020). For instance, Peters and Büchel (2010) found that functional coupling of the ACC and hippocampus as well as amygdala predicted the degree to which EFT reduced discounting rates. Therefore, the mediating role of the hippocampus in the relation between EFT ability and delay discounting may be through its role in mentally simulation potential future outcomes.

In the present study, we demonstrated that individual differences in EFT ability are associated with differences in delay discounting, and hippocampus may be an anatomical biomarker for this association. Our results suggest that the EFT and its associated brain structure in hippocampus may be critical factors that determine the differences in delay discounting. This is of particular interest as delay discounting is known to be a transdiagnostic indicator in psychiatric disorders (Amlung et al., 2019) and an important marker of impulsivity in addiction disorders (Pritschmann et al., 2021). Findings of our study perhaps provide evidence for interventions that improvement in the EFT ability can reduce steep delay discounting. However, several limitations of the current study should be borne in mind. Our study was cross-sectional in design and therefore could not address the causal relationship between EFT ability and delay discounting. Our hypothesis is that variation in EFT ability and associated brain structure leads to difference in delay discounting, but it is also possible that individuals’ intertemporal preferences shape their EFT ability. In addition, unlike most modified intertemporal tasks in which there are EFT cues and control trials, the present study had no control task, which results in our inability to see how specific these brain volume associations are to EFT and discounting. In addition, we focused on the volumetric features of gray matter that is a function of cortical thickness and surface area (Rakic, 1995). Volumetric techniques might obscure the extent to which each factor contributes to our findings. Finally, the participants in this study comprised a group of heathy young adults with a narrow age range, which may limit the generalizability of our findings. Future studies are needed to validate our findings using various morphometric methods and should extend the sample to a more diverse population, such as clinical populations, older adults.

In conclusion, we found that the EFT ability predicts delay discounting in young adults, and higher scores on EFT ability are associated with lower discounting. Furthermore, VBM results showed that GMV of two clusters in hippocampus and rACC significantly correlated with EFT ability, and the hippocampal GMV plays a significant mediating role in the association between EFT ability and delay discounting and might be the structural basis underlying this association. Our results suggest that the EFT ability might be an important factor that shapes people’s intertemporal preferences, and highlight the critical role of hippocampus in explaining the association of EFT ability and delay discounting.

## Data availability statement

The raw data supporting the conclusions of this article will be made available by the authors, without undue reservation.

## Ethics statement

The studies involving human participants were reviewed and approved by Ethics Committee of the Southwest University. The patients/participants provided their written informed consent to participate in this study.

## Author contributions

YG: project administration, conceptualization, formal analysis, investigation, methodology, validation, visualization, writing—original draft, and writing—review and editing. HW: conceptualization, formal analysis, writing—original draft, and writing: review and editing. ZL: conceptualization—writing—review and editing. LZ: writing—review and editing. TF: conceptualization, funding acquisition, project administration, supervision, and writing—review and editing. All authors contributed to the article and approved the submitted version.

## Funding

This study was funded by the National Natural Science Foundation of China (nos.32000777, 31971026, 61571070, and 62171073), the Scientific research Funds of Chongqing University of Posts and Telecommunications (no.E010A2018130), and the Guangdong Education and Science Project of the 13th Five-Year Plan (no.2018GXJK238).

## Conflict of interest

The authors declare that the research was conducted in the absence of any commercial or financial relationships that could be construed as a potential conflict of interest.

## Publisher’s note

All claims expressed in this article are solely those of the authors and do not necessarily represent those of their affiliated organizations, or those of the publisher, the editors and the reviewers. Any product that may be evaluated in this article, or claim that may be made by its manufacturer, is not guaranteed or endorsed by the publisher.

## Footnotes

^1^ http://www.fil.ion.ucl.ac.uk/spm

^2^ http://dbm.neuro.uni-jena.de/cat12/
